# MicroRNA Nano-Shuttles: Engineering Extracellular Vesicles as a Cutting-Edge Biotechnology Platform for Clinical Use in Therapeutics

**DOI:** 10.1186/s12575-024-00241-6

**Published:** 2024-05-21

**Authors:** Nico G. Menjivar, Jaiden Oropallo, Samuel Gebremedhn, Luca A. Souza, Ahmed Gad, Christian M. Puttlitz, Dawit Tesfaye

**Affiliations:** 1 Animal Reproduction and Biotechnology Laboratory (ARBL), Department of Biomedical Sciences, College of Veterinary Medicine and Biomedical Sciences, Colorado State University, Fort Collins, CO 80523 USA; 2 Orthopaedic Bioengineering Research Laboratory (OBRL), Translational Medicine Institute (TMI), Department of Mechanical Engineering, Colorado State University, Fort Collins, CO 80523 USA; 3 Orthopaedic Research Center (ORC), Translational Medicine Institute (TMI), Department of Clinical Sciences, College of Veterinary Medicine and Biomedical Science, Colorado State University, Fort Collins, CO 80523 USA; 4 J.R. Simplot Company, 1099 W. Front St, Boise, ID 83702 USA; 5 Department of Veterinary Medicine, College of Animal Science and Food Engineering, University of São Paulo, 225 Av. Duque de Caxias Norte, Pirassununga, SP 13635-900 Brazil; 6 Department of Animal Production, Faculty of Agriculture, Cairo University, Giza, 12613 Egypt

**Keywords:** Extracellular vesicles, miRNAs, Cargo carriers, Drug-delivery, Cancer, Therapies, Clinical studies

## Abstract

Extracellular vesicles (EVs) are nano-sized, membranous transporters of various active biomolecules with inflicting phenotypic capabilities, that are naturally secreted by almost all cells with a promising vantage point as a potential leading drug delivery platform. The intrinsic characteristics of their low toxicity, superior structural stability, and cargo loading capacity continue to fuel a multitude of research avenues dedicated to loading EVs with therapeutic and diagnostic cargos (pharmaceutical compounds, nucleic acids, proteins, and nanomaterials) in attempts to generate superior natural nanoscale delivery systems for clinical application in therapeutics. In addition to their well-known role in intercellular communication, EVs harbor microRNAs (miRNAs), which can alter the translational potential of receiving cells and thus act as important mediators in numerous biological and pathological processes. To leverage this potential, EVs can be structurally engineered to shuttle therapeutic miRNAs to diseased recipient cells as a potential targeted ‘treatment’ or ‘therapy’. Herein, this review focuses on the therapeutic potential of EV-coupled miRNAs; summarizing the biogenesis, contents, and function of EVs, as well as providing both a comprehensive discussion of current EV loading techniques and an update on miRNA-engineered EVs as a next-generation platform piloting benchtop studies to propel potential clinical translation on the forefront of nanomedicine.

## Introduction

The field of EV research has grown exponentially in recent decades due to their functional association as pertinent nano-shuttles to transfer bioactive molecules [1]. Extracellular vesicle, is an all-encompassing term underwritten by the International Society for Extracellular Vesicles (ISEV) to broadly connect lipid-encapsulated, secreted cellular particles to include exosomes, microvesicles (MVs), and apoptotic bodies (ApoBDs) [2, 3]. From the perspective as cargo carriers, EVs are intriguingly similar to liposomes, paralleling their dense phospholipid nature. Of specific distinction, dependent upon their biogenesis, EVs are constructed with a blend of lipids and surface membrane proteins, ultimately aiding in their downstream functions [4]. As tags for precise sites both locally and distant, intracellular molecules entertain the capacity to traffic through extracellular spaces, as effective drug carriers for therapeutic applications and novel scientific research avenues at the forefront of discovery.

Here, we discuss effective loading techniques to precisely harness EVs with miRNAs as bioactive compounds for the application as a cutting-edge platform for drug discovery and delivery. Previous attempts to review miRNA-enriched EVs focused primarily on the composition of EVs and the functional basis of miRNAs as future therapeutic prospects [5]. In this review, we largely focus on mechanisms of targeted loading miRNAs into EVs, with a principal element of incorporating recently published and impactful articles that include functionally relevant preclinical, clinical, and therapeutic involvement of engineered and/or modified EVs with specific nano-medicinal application.

## Biological role and EV uptake

### EV Biogenesis

Extracellular vesicles possess heterogeneous structural and biochemical properties, which reflect their cellular origin and biogenesis pathways. According to their origin, biogenesis, shape, and molecular constituents, EVs are broadly categorized as exosomes, microvesicles, and apoptotic bodies [6]. Exosomes comprise the smallest and rather homogenous size of EVs with a range in diameter from 30-150 nm and originate from the inward invagination of late endosomal membranes forming multivesicular bodies (MVBs), released into the extracellular space as exosomes upon fusion with the plasma membrane [7, 8]. Conceptually, during EV biogenesis, early endosomes are formed from the inward budding of the plasma membrane and mature to form intraluminal vesicles (ILVs) and MVBs. Frequently, the lysosomal fusion of MVBs results in its degradation. However, when MVBs contain CD63 in conjunction with lysosome-associated membrane proteins (LAMPs; LAMP1 and LAMP2), as well as MHC class II molecules, their contents are then released into the extracellular space upon fusion with the plasma membrane [9]. The formation of ILVs and MVBs are largely commissioned by the endosomal sorting complex required for transport (ESCRT)-dependent [10] or the ESCRT-independent pathways via the tetraspanin protein, CD63 [11]. Alternative pathways regulating ILV and MVB formation are associated with the sorting of cargo into exosomes [12]. On the other hand, microvesicles are often termed as shedding vesicles being comparatively irregularly shaped and a relatively heterogeneous population, with a size ranging from 100-1000 nm in diameter and generated via the outward budding of the plasma membrane [13]. Nevertheless, the largest fraction in size of EVs are apoptotic bodies, which range from 1–5 µm in diameter and are released during cellular disintegration preceding apoptosis [14, 15]. In addition to differing from their modes of biogenesis, EVs collectively can be differentiated based on their encapsulated molecular cargo contents. For instance, exosomes and MVs are more commonly preferentially enriched with a multitude of cytoplasmic components, including RNAs, proteins, and lipids. Alternatively, ApoBDs are largely enriched with cellular organelles and nuclear components [16, 17].

Ensuing biogenesis, EV release into the surrounding extracellular space is predominately facilitated by several subclasses of Rab family-GTPase proteins including RAB11, RAB35, and RAB27. For instance, exosomes are released from MVBs upon fusion with the plasma membrane through the facilitation of the RAB35 protein [18]. Moreover, exosomes are enriched with specific proteins including Wnt, PLP, TfR and flotillin [19]. The presence of highly-conserved and identifiable proteins, primarily membrane-associated proteins such as CD63, CD81, CD9, Alix, and TSG101 encompass hallmark characterization probes anchored amidst EVs [20]. These proteins also serve as marker proteins during EV processing and verification within EV preparations for functional studies as set forth by the guidelines in the Minimal Information for Studies of Extracellular Vesicles (MISEV), a position statement of the ISEV [2, 3]. All in all, crucial factors including the type and physiological status of varying cell types, ultimately determine facets of EV biogenesis, affecting their selection and packaging of key regulatory proteins [4].

### Unpacking EV cargo

In their infancy, EVs were assumed to be merely mediators that shuttle cellular toxicants into the extracellular space for the maintenance of cellular fitness and homeostasis [21]. However, studies have more recently shown the critical role of these nanovesicles in facilitating communication with neighboring cells, which are capable of posing both beneficial and deleterious effects. Phenotypic changes undergone by naïve recipient cells arise from the transfer of functionally active biomolecular components including lipids, proteins, mRNAs [22], and miRNAs [23], that subsequently interact with the ensuing extracellular matrix of naïve cells. Initial attempts to profile the protein contents of EVs have revealed their capacity to conceal both integrated proteins and proteins attached to their bilipid membranes. Although there is a clear divergence in the biogenesis pathways of both exosomes and MVs, no distinctive proteins have been reported that clearly differentiate the two EV subpopulations. This could in part be a result of the shared features of the endosomal and plasma membranes. Reports have shown that proteins necessary for exosome biogenesis such as ESCRT proteins are highly abundant in the proteome profile of exosomes [24]. Due to the amplified nuclease activity amidst the extracellular environment, it can be assumed that secreted EVs are potentially vulnerable and subject to degradation. Therefore, EV structural composition is integral in protecting their harnessed bioactive cargo from degradation upon release into the extracellular space [25]. The multifaceted structural characteristics that allow EVs to withstand adverse extracellular conditions poise them as attractive vehicles to shuttle therapeutic proteins and RNAs as a potential drug delivery platform [26, 27]. Various forms of EV interactions with recipient cells underpin their functional delivery of bioactive molecules [28]. Once integrated within recipient cells, EVs are capable of evading lysosomal degradation as a means to release their functional cargo molecules. Here we focus on miRNAs, which exert functional regulatory impacts on gene expression through post-transcriptional regulation of target mRNAs. It is well-documented that the expression level of particular miRNAs are key during development [29, 30] and stress responses [31, 32], and that EVs play an important role in transferring miRNA cargoes between cells [33]. Alterations in the release and uptake of EVs are associated with pathologies including cancer [34] and cellular stress [32, 35–39].

### Mechanisms of EV uptake

The uptake of EVs by recipient cells can be confirmed either via direct or indirect evidence. EVs can be directly visualized by labeling their bilipid membranes using lipophilic fluorescent dyes, including the commonly used PKH67 and PKH26, Rhodamine B (R18) and DIL [40]. Alternatively, EVs can be stained using permeable dyes like CFSE and CFDA, amidst the confines of their cytoplasmic lumen [41]. The incorporation of fluorescently labeled EVs can then be verified either using microscopy [42] or via cellular sorting technologies in recipient cells using flow cytometry [28, 40]. In vivo, various mechanisms of EV internalization into recipient cells and/or tissues are discussed within the literature across many fields of study. Among those mechanisms commonly represented, clathrin-mediated endocytosis, phagocytosis, micropinocytosis, lipid raft-mediated internalization, and direct fusion with the plasma membrane of the receiving cell are among the most widely reported. Specific physiological uptake mechanisms of EVs are largely dependent upon their molecular composition, most commonly the surface protein and/or glycoprotein configurations of both the EVs membrane and the plasma membrane of the receiving cell [43]. This has in part been confirmed where tetraspanin proteins CD9 and CD81, which are present on the surface of EVs, were evidenced to play important roles in the cellular uptake of EVs. Cells treated with anti-CD9 and anti-CD81 antibodies showed similar reductions in EV uptake [44]. Additionally, a study using EVs treated with proteinase K (broad-spectrum serine protease used for protein digestion), resulted in the reduced uptake of EVs in recipient cells [45]. Conceptually, the dominant mechanisms of EV uptake can vary greatly amongst cells of differing pathophysiological conditions.

## Extracellular miRNAs and their incorporation within EVs

### miRNAs

**Fig. 1:**
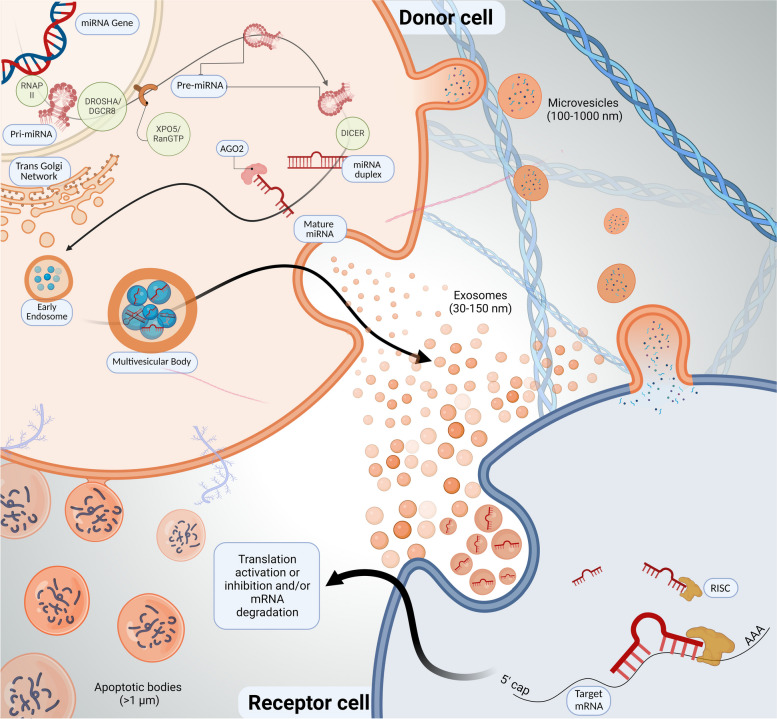
Diagrammatic Overview of Extracellular Vesicle Biogenesis and miRNA Processing. Extracellular vesicles (EVs) are a heterogeneous collection of membrane-enveloped nanoparticles that serve as a mass transit mechanism for the packaging and release of complex cargos, including miRNAs. Exosomes (30-150 nm), are commonly spherical in shape and arise via the endocytic pathway via exocytosis from the fusion of the vesicular membrane into the plasma membrane. Microvesicles (100-1000 nm), notably irregularly shaped, are the byproduct of the outward budding/pinching of the plasma membrane. Apoptotic bodies (> 1 µm) are formed through apoptotic cell disassembly or programmed cell death, and released through cell blebbing. The cargo 'selection' or 'sorting' process that ensues, specifically the local enrichment of miRNA cargo molecules during nascent EV formation is largely propagated through the miRNA processing enzymes Drosha and Dicer, required for the maturation of miRNAs that lead to the translational repression or degradation of target mRNAs

As implied by their name, miRNAs are short, single-stranded RNA molecules of ~ 22 nucleotides, initially discovered by Lee and colleagues in 1993, while studying the nematode *Caenorhabditis elegans* [46]. MicroRNAs function to effectively modulate the stability of mRNA, most commonly inhibiting the translational potential and/or inducing degradation to respective mRNA targets via a sequence-specific complementarity mechanism [47, 48]. Focusing primitively on canonical miRNAs, their biogenesis primarily initiates from DNA sequences called miRNA genes, which are then transcribed into primary miRNAs (pri-miRNA; ~ 150 nt) by RNA polymerase III, and further processed via a microprocessing system into precursor miRNA (pre-miRNAs; ~ 70 nt). Pre-miRNAs are then shuttled from the nucleus of the donor cell into the cytoplasm via a complex of exportin5 and RAS-related nuclear protein-guanosine-5’-triphosphate-ase [49]. Amid the cytoplasm, the terminal loop of pre-miRNAs are sequestered via the RNase III endonuclease, Dicer [50], molding miRNA duplexes that are then catalyzed by Argonaute RISC Catalytic Component 2, responsible for leaving and/or removing one strand of the duplex to propagate the directionality of a mature miRNA strand [51, 52] with the capacity to be packaged into EVs for potential functional alterations upon its effective release amidst receptor cells (Fig. 1).

Initial studies reporting that EVs carry miRNAs [23, 53, 54] have fueled further research efforts to unpack the all-inclusive nature of EVs and their biological cargoes. It has been previously reported that over 60% of all mammalian mRNAs are predicted to be post-transcriptionally regulated by miRNAs [55], indicating that miRNAs functionally constitute a significant class of pervasive regulators of various cellular processes, outnumbering kinases and phosphatases [56]. Collated data generated from small RNA sequencing reports have indicated that miRNAs comprise anywhere from < 1% to 30% of the total read counts within EVs of diverse origins [57]. Extracellular miRNAs encapsulated within EVs are progressively being explored as promising circulating biomarkers for many cancers and diseases [58], and their ability to remain predominantly stable while evading degradation from external nucleases, underpins their significance, and the basis for studying EVs as cargo carriers for downstream therapeutic usage. However, the precise mechanisms by which specific miRNAs are packaged and released and/or enriched into EVs still largely remains unknown.

### Encasing selective miRNAs into EVs

Attempts to pinpoint the explicit processing mechanisms leading to extracellular miRNAs export into EVs versus their cellular retention have been predominantly inconclusive. Many studies exist to suggest that collectively, RNAs are primarily shuttled into EVs selectively via the interaction of specific RNA sequence motifs [59] or lipid interactions [60] in association with RNA-binding proteins (RBPs) [61–63], or possibly through non-selective measures as evidenced previously [64, 65]. In more recent efforts and extending past the initial observations of single tetranucleotide motifs connected to miRNA export [61, 66, 67], Garcia et al. has shown up to an 80-fold enrichment of specific RNA sequence motifs (CGGGAG) identified by ‘reader’ proteins Alyref and Fus, which function to promote the sorting of sequence motif-bearing miRNAs into EVs for potential downstream delivery [59]. Still, a range of other EV-sorting signals including RNA and/or RBP modifications that inherently impact RNA stability and miRNA biogenesis also exist [68], such as ubiquitylation, sumoylation, phosphorylation, and uridylation, which likely involve regulatory processing machinery that implicate miRNA packaging into EVs. Together, these studies along with many others suggest an overabundance of influences that likely labor interchangeably, and coalesce in the packaging of various forms of RNAs into EVs. A more detailed understanding of RNA incorporation mechanisms is discussed in a recent review by Dellar and colleagues [57].

## Methods of miRNA cargo loading for incorporation into EVs

Primitive compositional and nano-mechanical properties of EVs including their admirable biocompatibility and stability, non-cytotoxic and low immunogenic traits, high loading ability and lengthy life span, and their intrinsic aptness to cross biological barriers make them ideal drug delivery candidates that natively carry cargo components, easily modifiable to contain therapeutic agents of interest (*e.g.*, nucleic acids). Recent evidence in mice using engineered EVs with small interfering RNAs (siRNAs), indicated more than a tenfold improvement in functional siRNA delivery in contrast to synthetic lipid nanocarriers [69]. Compared to EVs, to date, a multitude of hindrances exist in developing synthetic nanocarriers for downstream clinical usage in drug delivery [70], specifically involving their toxicity and immunogenic responses, lack of specificity, and preferential aggregation amidst the liver and spleen [71]. All things considered, copious evidence suggests that the advantageous and distinct features of EVs are likely the eminent angle catalyzing their integration as a mainstream effort at the forefront of nanomedicinal discovery.

**Fig. 2:**
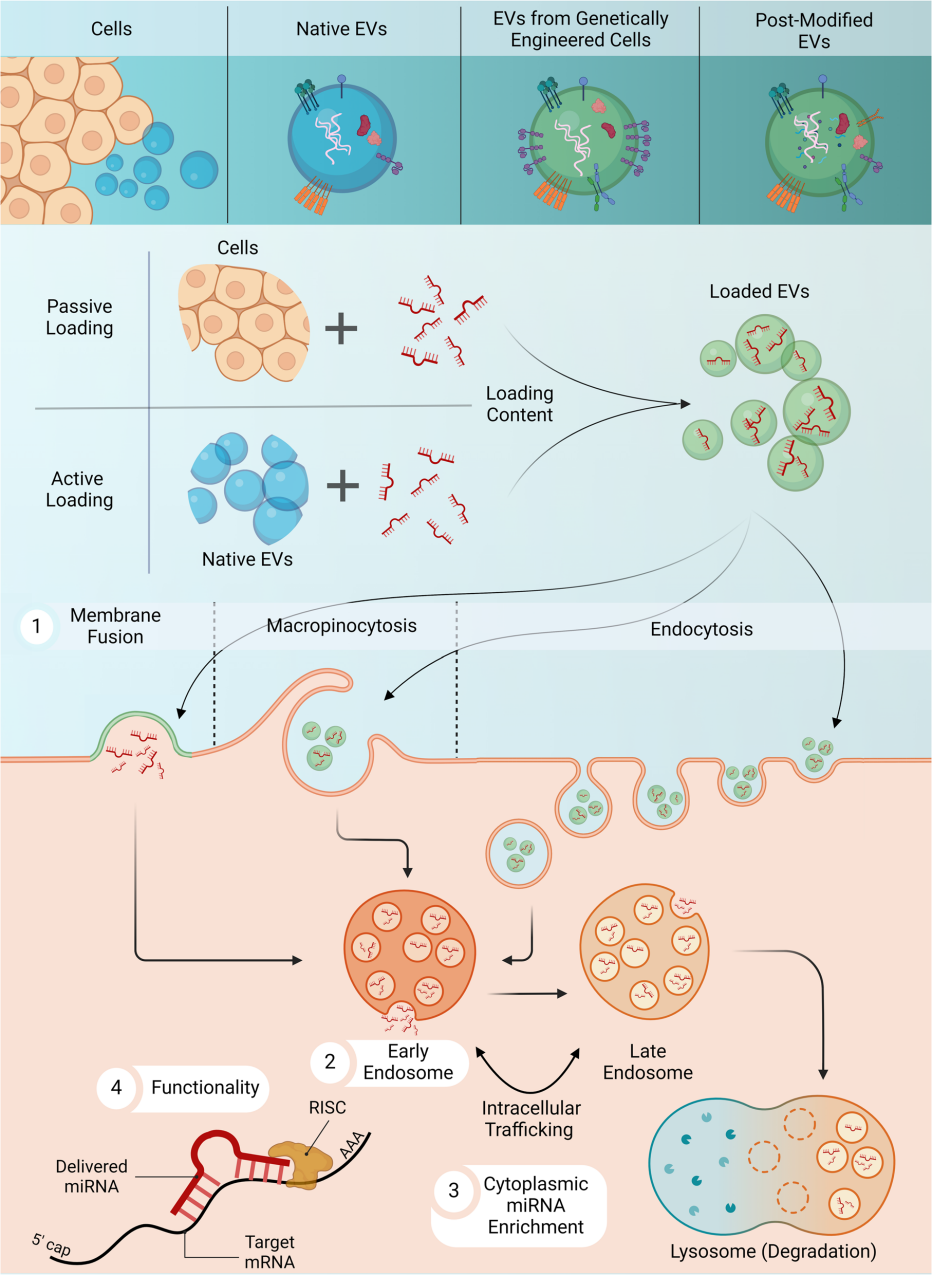
Schematic Representation of Methodological Approaches in Extracellular Vesicle Loading for Functional Uptake in Target Cells. Lipid bilayer-delimited particles (EVs) serve as an effective novel drug delivery system for endless pharmaceutical compounds, including miRNAs. Thus, the method of incorporation for enriching miRNA cargoes into EVs can be segmented into two main sub-types: passive (donor cell manipulation) and active (direct EV alterations) loading methods

Complexities in EV sample heterogeneity combined with the variability in encapsulated molecular cargoes primitively pose an inherent need in EV loading mechanism optimization in producing cargo-modified EVs for downstream therapeutic applications. Conceptually, EV loading techniques can be categorized into two main approaches: indirect modification to donor cell physiology (‘endogenous’ or ‘passive’ cell-based alterations) or through the direct modification of EVs (‘exogenous’ or ‘active’ cargo harnessing), each of which, with varying degrees in efficiency. Utilizing ‘endogenous’ or ‘passive’ loading measures, molecular constituents act upon and are taken up via donor cells, and subsequent excess cargo can then be shuttled into EVs prior to formation, resulting in the subsequent secretion of indirectly modified EVs with an increased abundance of the molecular component of interest. Assuming a more natural role in cargo loading, the enriched EVs can then be utilized as a delivery platform to recipient cells [72]. Alternatively, ‘exogenous’ or ‘active’ loading measures, primarily draw a focus on implementing catalytic reagents post-EV isolation to induce a permeable bilipid membrane to bolster cargo loading with precision molecules of interest [73]. In spite of the fact it is a more direct approach, such re-engineering of EVs heavily influences the composition of their bilipid membrane, which serves as the primary contact point in cell-to-cell communication propagating the various mechanistic routes of their ensuing uptake [74] (Fig. 2). The following sections offer a more comparative approach of multiple loading techniques in greater depths, focused on cargo loading efficiency with an emphasis on loading selective miRNAs into EVs.

### Endogenous (Passive) pre-loading of EVs

Pre-loading miRNAs as potential therapeutic cargoes into EVs is a method widely executed across many disciplines, largely accomplished through donor cell manipulation via incubation and transfection with synthetic miRNAs (also known as miRNA mimics) and/or with miRNA-expressing plasmids/viral vectors, to increase endogenous levels of selective miRNAs that can then be passively incorporated into EVs during their biogenesis [75]. Although, current attempts in manipulating donor cells to secrete therapeutic-miRNA enriched EVs largely bypass the structural and/or compositional alternations of miRNA loading post-isolation, recent reports also suggest that circumjacent transfection reagents critically affect downstream RNA cargo delivery [76–78]. Nevertheless, methods of endogenous cargo loading are still widely used, warranting further discussion of specific loading techniques.

#### Donor cell manipulation and genetic engineering

Direct transfection of EV-secreting cells as a means to modulate their cargo content is a rather straightforward and simple method to enrich or deplete EVs, prior to their inception. The use of chemical transfection reagents to load synthetic mimics and/or precisely designed vectors/plasmids effectively transduced into donor cells, are the two predominant methods utilized in overexpressing desired molecules (therapeutic drugs), such as small nucleic acids to include miRNAs. Assimilating and concentrating miRNAs within the cytosol of naïve cells following assisted passage across the cell defining plasma membrane, miRNAs have the potential to escape the endolysosomal system, and withstand dynamic and degradative ribonucleases. If successful, assimilated miRNAs typically favor incorporation into ILVs, which foregoes their exocytosis within EVs. Endogenous RNA modulations have been reported using commercialized transfection reagents such as Lipofectamine™ 2000 (Thermo Fisher Scientific; Waltham, MA, USA) and HiPerFect® (Qiagen; Hilden, Germany), artificially designed constructs/vectors [79], RNA aptamer–protein interactions and reversible light-inducible protein–protein interaction modules [80], EXOtic RNA-packaging devices [81], and the TAMEL loading platform [82]. To this point, the generation of stably modified HEK293T cell lines designed to express miR-21, have been reported to produce modified EVs for downstream therapeutic use in glioblastoma rat models [83]. In addition, the development and delivery of CRISPR-Cas9 technologies as a novel gene editing molecular tool has also been shown to be applied to living cells through electroporation for the establishment of manipulated parent cells designed for the production of EVs bearing specific cargoes [84–86]. Although extensive damage to the physiochemical properties of EVs using passive loading measures is largely averted, preserving their integrity, the endogenous pre-loading of EVs is predominantly unmanageable due to the elusive disparities in RNA incorporation within EVs [63], and the presence of residual contaminants such as transfection reagents that ultimately affect RNA cargo delivery [76–78]. Given the regulatory properties of miRNAs, the low loading efficiencies of this method likely induce some level of cytotoxicity in donor cells, promoting ensuing cellular damage that hinders a natural homeostatic condition, thus altering the function of subsequent EV secretions under basal conditions of homeostasis [87, 88]. Moreover, a major drawback of this approach is the lack of control over the incorporation of other cargoes, including non-selective miRNAs, mRNAs, lipids, and proteins. Furthermore, the contribution of specific miRBPs in functionally regulating in part the efficiencies of passive loading of target miRNAs into EVs, is not yet fully investigated or understood, ultimately preventing a more specific and controlled method of cargo loading.

### Exogenous (Active) post-loading of EVs

Post-isolation loading of EVs with exogenous biomolecules has been principally achieved through incubation strategies promoting a close-knit association of the cargo of interest with the EV lipid bilayer membrane [89], harnessing therapeutic cargo interests to the EVs surface [90], or most regularly through the diffusion of biomolecules into EVs employing techniques to mechanically and/or chemically stimulate a porous membrane [91]. Despite initial reports indicating a low cargo loading efficiency and an ineffective delivery of active RNA cargos [92], more recent reports have shown a robust miRNA upregulation (> 1000-fold) into EVs via exogenous manipulation [93]. However, mechanistic follow-up studies to evaluate the compositional surface of EVs in supporting the delivery of targeted cargo, effectively need to be further elucidated to unlock the full translational potential of EVs as carriers of therapeutic agents. The following subsections review precisely in greater depth, common methods of exogenous cargo loading, elucidating both their strengths and drawbacks as a serviceable EV loading system.

#### Provisional membrane permeabilization

Understanding the compositional elements encompassing the structure of EVs is quintessential to conceptualizing progressive approaches for therapeutic alterations in cargo packaging. Broadly speaking, bi-lipid encapsulated nanoparticles (EVs) principally express surface ligands and receptors from their source cells, all while encircling a hydrophilic core [94]. Early attempts of miRNA cargo loading into EVs post-isolation, all followed a common and relatively simple method of incubation. Under specialized conditions, cationic lipid formulations like Lipofectamine™ RNAiMAX Transfection Reagent (Thermo Fisher Scientific; Waltham, MA, USA) have been shown to successfully incorporate miR-335-5p into EVs for functional therapeutic delivery in vivo to desmoplastic cancers [95]. Pending the cargo of interest, incorporation into isolated EVs is targetedly achieved through simple diffusion across the EVs bilipid membrane, while its stunted loading efficiencies are generally restricted via the concentration gradient within the solution and the hydrophobicity of the loading compounds [91]. Other direct mechanistic approaches to destabilize the bilipid membrane of EVs are predominantly performed through sonication and electroporation. For instance, recent reports from Pottash et al. used sonication-mediated EV loading techniques to incorporate anti-inflammatory miRNAs (miR-146a, miR-155, and miR-223) into HEK293T EVs for downstream use in inflammation-related diseases [96]. The focus of sonication is on disrupting the membrane rigidity and microviscosity through ultrasound waves, while the electroporation of EVs is highly dependent upon their subjection to a high-voltage pulsing that dismembers its pores. Taken together, central technical adversities exist involving both methods including the destabilization of the membrane and the preservation of EV integrity, which regulate and affect downstream cellular uptake [97]. Additionally, the electroporation of EVs with uncommitted nucleic acids have been described to manifest large cargo aggregates, which have partially been attempted to have been offset by incorporating EDTA in conjunction with an electroporation buffer [98]. Aside, recent reports have suggested that sonication-assisted loading (28%) is comparably more efficient than both incubation (1%) and electroporation (5%), respectively [99].

Various other techniques exist to include: calcium chloride transfection, freeze–thaw cycles, pH gradient modifications, as well as kit-based assays like the Exo-Fect™ siRNA/miRNA Transfection Kit (System Biosciences; Palo Alto, CA, USA) that secure the potential application to targetedly load precision molecules into EVs as a new and promising frontier among established drug delivery systems. Exogenous loading of miRNA molecules using calcium chloride transfection has been evidenced and slightly modified via heat shock, which alters the fluidity of the exosomal membrane to promote the incorporation of miRNA into EVs with similar efficiencies in loading to electroporation [100]. Additionally, the notion of EVs as nanocarriers has also been evidenced through the successful accumulation of carrier RNA complexes using repeated freeze–thaw cycles (10 times), from room temperature to -80 °C [101]. Although, recent evidences have duly suggested that repeated freeze–thaw cycles leads to a reduction in the number of EVs, as well as a cycle-dependent increase in their particle size, suggesting the phenomena of EVs subsequent fusion during storage [102]. Aside, given the structure and composition of EVs, pH gradients between the intravesicular and extravesicular environments have also been studied to mechanistically load negatively charged cargos via dehydration/rehydration using 70% ethanol and acidic citrate buffer (pH 2.5) followed by dialysis in HEPES-buffered saline (HBS; pH 7), respectively. The same study revealed decreased levels of Alix and TSG101 following mechanistic measures of pH gradient modifications, suggesting potential surface protein and lipid rearrangements amid the EVs surface [103]. Of particular ineterst in terms of loading efficiencies, recent reports have indicated that EV cargo modulations via the Exo-Fect™ system (System Biosciences; Palo Alto, CA, USA) have revealed a > 1000-fold upregulation of specific miRNA moleculaes of interest. In the same study, it was also shown that Exo-Fect™ miRNA-modified EVs contained altered membranes that catalyzed their internalization within target cells, while duly minimizing their lysosomal colocalization compared to native EVs, underpinning a very promising method of incorporation for loading therapeutic miRNAs into EVs [93]. These summarized findings elucidate useful technical advancements in developing an efficient platform to produce therapeutic miRNA-enriched EVs capable of downstream clinical usage.

## Therapeutic and clinical translation of miRNA Cargo-Enriched EVs for disease treatment

**Fig. 3:**
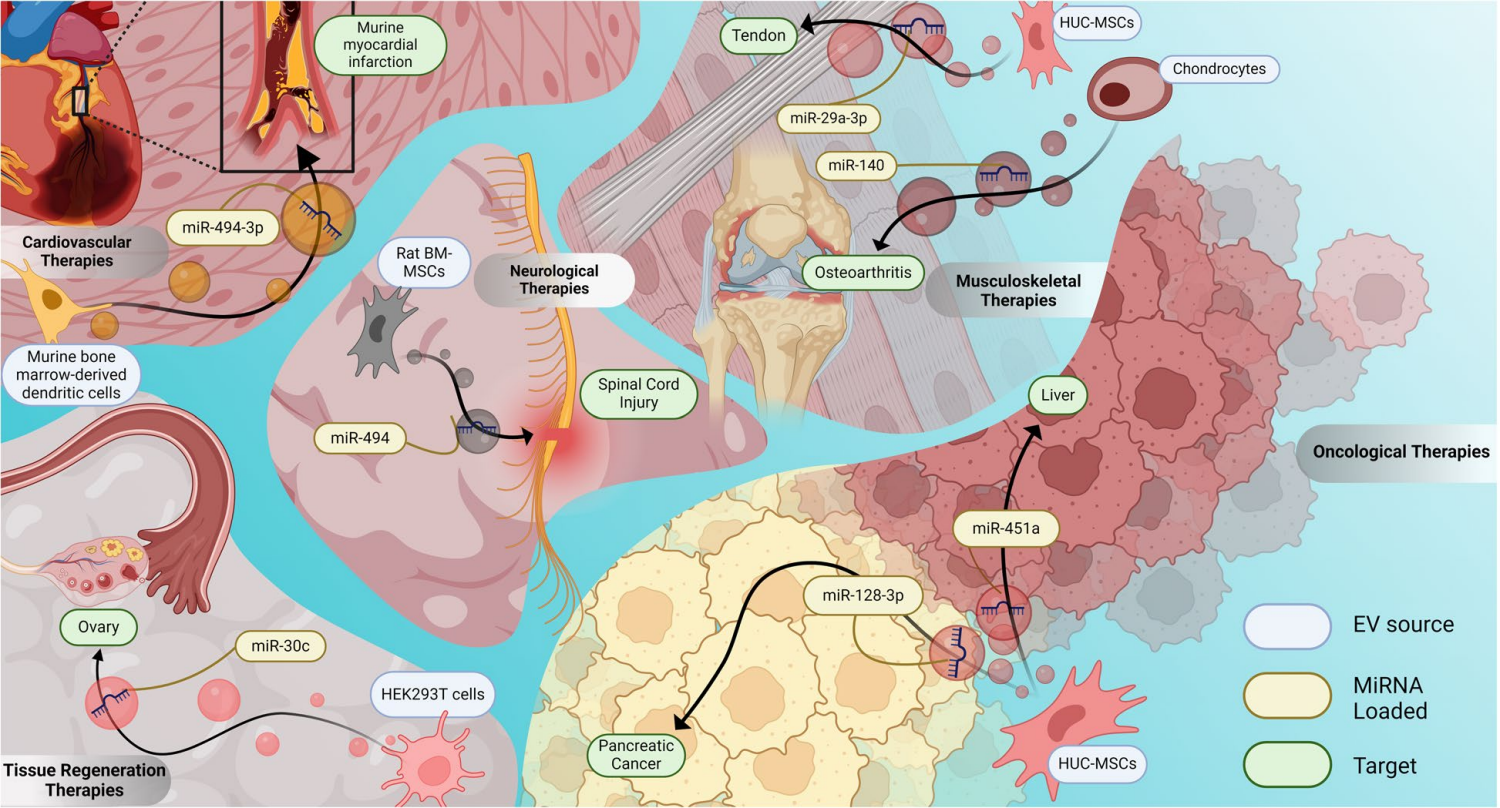
Conceptual Illustration of EV-miRNAs Regulatory Role in Intercellular Communication Among Selected Reviewed Studies. Collectively, EV treatments have been implicated as a targeted approach in combating disease, as therapeutically-enhanced tiny particles with immense therapeutic potential in nanomedicine. Major areas of therapeutic interest include: tissue regeneration, oncology, as well as within various musculoskeletal, cardiovascular, and neurological disorders

EVs hold great therapeutic and clinical potential, owing partially to their ability to be used in allogeneic and/or xenogeneic applications without eliciting signs of cytotoxicity and/or immune reaction [104–106]. As such, they are routinely investigated as both a stand-alone therapy and as carriers for a variety of drug-based therapeutic compounds. Of these applications, miRNA loading into EVs has gained significant popularity within the last decade, given the proven regulatory properties of miRNAs becoming more widely recognized within a variety of tissues [107]. The first results of miRNA loading into EVs quickly followed such discoveries, with Katakowksi et al. 2013 demonstrating that engineered mesenchymal stem cell (MSC) exosomes loaded with miR-146b, having the ability to significantly reduce glioma xenograft growth within a rat model of primary brain tumor [108]. That same year, Ohno et al. adapted measures to transfect HEK293 cells with Let-7a, revealing that the purified exosomes resulting from the cell culture were able to effectively, translationally inhibit tumor formation in a murine model of xenograft breast cancer [109], while Bryniarski et al. duly used exosome-like nanovesicles to deliver miR-150, as a means to regulate T-cell tolerance, which also in a murine model showed to inhibit allergic contact dermatitis [110]. Agreeingly, these discoveries provided early evidence of the therapeutic application and clinical potential, which has since aided in helping to shape the steadily increasing interest in miRNA-enriched EV therapies spanning multiple disciplines, as evidenced in the provided schematic (Fig. 3).

Recent research into the clinical application of miRNA cargo-enriched EVs have been heavily driven by their oncological appeal, notably in breast, lung, liver, colorectal, ovarian, and brain tissues, given that cancer progression and metastasis are intently connected with the repressed expression of tumor-suppressing miRNAs [111]. In addition, the therapeutic applicability of miRNA-enriched EVs are under investigation regarding tissue regeneration and amelioration of chronic illnesses in nearly every tissue in the body (Table 1); most prominently in the musculoskeletal, cardiopulmonary, and nervous systems. Research studies involving the potential clinical translation of miRNA-enriched EVs encompasses a large body of literature/number of publications, spanning a lengthy list of high-impact scientific journals amidst diverse fields, previously summarized by other groups [5]. With the rapid growth of miRNA-enriched EV research enhancing our basic scientific knowledge of EVs for downstream application, this review largely focuses on previously undiscussed publications with the substantial translational potential to feasibly favor the journey from benchtop to clinic.

**Table 1:** miRNA-engineerd EVs as an advanced drug delivery system in *Tissue Regeneration Therapies*

Therapeutic miRNA	miRNA Enrichment	Incorporation method	Source of EVs	Target	in vitro Cell Lines Utilized	in vitro Findings	in vivo Model	in vivo Findings	Source
miR-21-5p	Exogenous	Electroporation	Human ADSCs	Diabetic cutaneous wound healing	HaCaT human keratinocytes	Promotion of cell proliferation and migration via Wnt/b-catenin pathway	Rat diabetic cutaneous wound model	Significant promotion of wound closure, increased re-epithelialization, and increases in both total blood vessel count and mature blood vessel count	Lv, Qijun, et al. [112]
miR-31-5p	Exogenous	Electroporation	Raw milk	Diabetic wound healing	HUVECs	Increased cell proliferation, migration, and angiogenesis	Rat diabetic cutaneous wound model	Decrease in unclosed wound rate, increased collagen deposition and re-epithelialization rate, and improved vascular network formation	Yan, Chengqi, et al. [113]
miR-30c	Endogenous	Transfection	Ectopic endometrial endothelial cells	Ovarian endometriosis	N/A	N/A	Murine endometrial model	Decrease in ectopic nodules and attenuation of metastatis via BCL9/Wnt/CD44 regulatory cascade	Zhang, Mengmeng, et al. [114]
miR-31-5p	Endogenous	Lentiviral Transduction	HEK293T cells	Diabetic wound healing	HaCaT human keratinocytes, HFF-1 human foreskin fibroblast, EA.hy926 human endothelial cells	Promotion of cell proliferation and migration; as well as increased capillary-like construction activity of endothelial cells	Rat diabetic cutaneous wound model	Increases in cutaneous wound closure rate, blood vessel density, and number of mature blood vessels, as well as increased collagen deposition	Huang, Jinghuan, et al. [115]

### Oncology

Globally, nearly 20 million people are diagnosed with cancer every year, with that number predicted to rise to nearly 30 million by 2040 [116]. Given the steady increase in the global cancer burden, it is imperative that new therapeutic strategies are developed to reduce cancer deaths. Trends in miRNA-engineered EV therapies for cancer treatment have catapulted studies amidst some of the most commonly diagnosed and deadliest cancers, with a strong focus on breast, lung, liver, and colorectal cancers. Significant research has also been conducted on ovarian and brain cancers, likely due to their high mortality rates [117].

Breast cancer has recently surpassed lung cancer as the most commonly diagnosed cancer, with an estimated 2.3 million new cases every year, globally [116]. As such, it has become the target of multiple potential miRNA-engineered EV therapies. Largely, targeted therapies for breast cancer can be bisected into two groups, with a focus either on the activation of the immune system to inhibit malignant growth or through the direct tumor suppression via miRNA-mediated cellular apoptosis. Case in point, a previously reported attempt using exosomes derived from M1 macrophages, loaded with miR-511-3p were opportunely surface-modified to include interleukin-4 receptor-binding peptide and subsequently showed to successfully induce M1 polarization through the downregulation of M2 markers in vitro, showing greater homing properties to tumors in a murine model using 4T1 breast cancer cells. In vivo experiments duly confirmed the inhibition of tumor growth and decreased levels of M2 cytokines and immune-suppressive cells, increasing levels of M1 cytokines and immune-stimulatory cells [118]. In addition, other groups have also reported the successful targeting of breast cancer cells with miRNA-loaded EVs, namely miR-34a-enriched exosomes, which were shown to dose-dependently induce breast cancer cell death, visibly increasing the inhibition of cell migration and invasion, compared to unloaded-exosomes [119]. Conversely, lung cancer, now the second most prevalent cancer worldwide, is reported as the leading cause of cancer death [116]. Implemented strategies to actively inhibit non-small cell lung cancer have been predominantly focused on promoting tumor cell apoptosis in both human cancer cell lines and murine models. For example, Zhou et al. tested the efficacy of miRNA-enriched exosomes on both A549 cells, as well as in a murine xenograft model. The application of miR-449a-loaded exosomes to A549 cells, revealed their ability to effectively inhibit proliferation and promote apoptosis, while duly decreasing tumor volume, nearly doubling the survival time of the mice in vivo [120]. Although these studies demonstrate the modulatory effects and the significance of selected candidate miRNAs as a targeted treatment to combat several types of aggressive cancers, further in-depth studies are required to explicitly evaluate their encompassing functions and off-target effects in order to ultimately apply these therapeutics to in vivo human models as EV-based miRNA therapies.

Apart from breast and lung cancers, liver cancers are currently the third leading cause of cancer-related deaths, accounting for approximately 8.3% of all cancer-related deaths worldwide [116]. Given miRNAs’ powerful use as both biomarkers and mediators of physiology and disease, miRNA-enriched EV therapies for liver cancers have predominantly focused on identifying candidate miRNAs downregulated in hepatocyte cancer progression, prior to designing potential strategic EV-based therapies. Yu et al. tested the anti-tumor capacity of lowly expressed miR-375 in human hepatocellular carcinoma (HCC), revealing miR-375-loaded exosomes ability to reduce proliferation, increase apoptosis, and decrease the number of migratory and invasive HCC cells in vitro. Employing the use of an in vivo murine model, the anti-tumor effects of miR-375-loaded exosomes injected with Huh-7 cells were shown to result in a significant inhibition of Huh-7 tumor growth, with reductions in the proportion of KI67-positive proliferative cells [121]. Alongside liver cancers inimical effects, colorectal cancer (CRC), the third most commonly diagnosed cancer and the second leading cause of cancer death [116], has also positionally implemented broad utilization of EV-based treatments using a numerous amount of cell lines for both in vitro and in vivo work. Alike, a representative study from Hosseini et al. aimed to investigate the anti-tumor effects of CT-26 murine CRC-derived exosomes loaded with miR-34a in an in vivo murine model, disclosing miR-34a-loaded-exosomes ability to greatly reduce tumor size, prolonging the survival time of mice. Additionally, miR-34a-enriched exosomes were shown to aptly induce T cell polarization towards the cytotoxic T cell subtype, which is in part responsible for targeting and destroying cancerous growths [122]. In addition to those aforementioned cancerous subtypes, ovarian cancer (OC), accounts for 5% of female cancer deaths and approximately 2.5% of all malignancies among women, despite its 1.3% lifetime risk of development in the United States [117]. Despite the immense amount of research in the field, a massive shortage persists for promising screening tools given OCs high morbidity and low survival rates. In the case of miRNA-engineered EV therapies, two predominant strategies are commonly utilized and explored for OC: (1) comparing the miRNA expression patterns between healthy and cancerous ovarian tissues aimed in identifying potentially therapeutic miRNAs, or (2) selecting miRNAs capable of reducing cancer cell sensitivity to chemotherapeutics. A previous attempt by Zhao et al. aimed to combine both approaches through first selecting a candidate miRNA (miR-484) post-comparison of the miRNA expression patterns of ovarian cell lines and tissues, denoting the significant downregulation of miR-484 in cancerous tissue types. Aimed to unveal its functional relevance in a murine model revealed, miR-484-loaded HEK293T-derived exosomes improved vascular normalization and the chemotherapeutic sensitization of ovarian tumors [123]. Collectively, these studies suggest the broad efficacy of identifying potential targeting therapies through the investigation of differentially regulated miRNAs amidst cancerous tissues, a technique that could be more broadly applied to create therapeutics for both cancerous and non-cancerous illnesses.

Alas, brain cancers, including gliomas and meningiomas, constitute fairly rare types of cancers, but otherwise have one of the lowest survival rates. Alongside, such cancers persist and are particularly prevalent in areas embodying a high Human Development Index (i.e. North America, Europe, Russia, China), composing double the incidence and mortality per 100,000 people, compared to areas with a low Human Development Index [116]. These statistics undeviatingly underpin just why brain cancers are a common target for miRNA-engineered EV therapeutics, with glioma treatments being actively investigated by multiple groups. From initial attempts screening glioma stem cell lines, Lang et al. found that miR-124a packaged into BM-MSC exosomes showed significant reductions in glioma stem cell viability and clonogenicity. Using an i*n vivo* murine model, glioma stem cell lines were exposed to miR-124a-loaded exosomes prior to intracranial xenograft, revealing a 50% survival rate over a 120-day period, whereas both the PBS and unloaded-exosome control treatments plummeted to a 0% survival rate by day 70 [124]. In short, initial piloting efforts in the development of cancer therapies for implementation in mainstream medicine by use of miRNA-engineered EVs are currently being explored both using in vitro and in vivo models, as an effective nanodelivery system (Table 2).

**Table 2:** miRNA-engineerd EVs as an advanced drug delivery system in *Oncology*

Therapeutic miRNA	miRNA Enrichment	Incorporation method	Source of EVs	Cancer Target	in vitro Model	in vitro Findings	in vivo Model	in vivo Findings	Source
miR-130	Exogenous	Electroporation	4T1 breast cancer cells	Breast	Peritoneal macrophages from female C57BL/6 mice	Repolarization of M2 macrophages to M1 phenotype; reprogrammed macrophages then showed the ability to reduce 4T1 breast cancer cell prolifertaion, migration, and invasion abilities	N/A	N/A	Moradi-Chaleshtori M, et al. [125]
miR-33	Exogenous	Electroporation	4T1 breast cancer cells	Breast	Peritoneal macrophages from female BALB/c mice	Repolarization of M2 macrophages to M1 phenotype	N/A	N/A	Moradi-Chaleshtori M, et al. [126]
miR-511-3p	Exogenous	Exo-Fect™	Murine M1 macropahges from bone marrow-derived monocytes	Breast	M2 macropahges and 4T1 breast cancer cells	Repolarization of M2 macrophages to M1 phenotype	Murine 4T1-luc xenograft model	Exosome homing to tumors, inhibition of tumor growth and increased presence of M1 marcophages and immune-stimulatory cells	Gunassekaran GR, et al. [127]
let-7i, miR-142, miR-155	Exogenous	Electroporation	4T1 breast cancer cells	Breast	Bone marrow-derived dendritic cells, T cells, and 4T1 cells	Promotion of dendritic cell maturation and increased T cell proliferation and colony formation around tumor cells	Murine 4T1 xenograft model	Significant reduction in tumor size and increase in percent survival over a 50 day period	Khani AT, et al. [128]
miR-34a	Endogenous	Lentiviral Transduction	Genetically modified dental pulp MSCs	Breast	MDA-MB-231 breast carcinoma cells	Breast cancer cell death in a dose-dependent manner and increased inhibition of cell migration and invasion	N/A	N/A	Vakhshiteh F, et al. 211
let7c-5p	Exogenous	Lipofectamine™ Transfection	HEK293T cells	Breast	MDA-MB-231 breast carcinoma cells	Inhibition of breast cancer cell proliferation and migration	N/A	N/A	Kim, Haneul, and Won Jong Rhee. [129]
miR-1296	Exogenous	Incubation/Rehydration	Nanoliposomes	Breast	MDA-MB-231 breast carcinoma cells	Significant reduction of cell viability in a dose-dependent manner and increased sensitization to cisplatin	N/A	N/A	Albakr, Lamyaa, et al. [130]
miR-3182	Exogenous	Electroporation	Human umbilical cord mesenchymal stem cells (HUCMSCs)	Breast	MDA-MB-231 breast carcinoma cells	Reduction of cell proliferation and migration and induction of apoptosis via the downregulation of the mTOR and RPS6K1 genes	N/A	N/A	Khazaei-Poul, Yalda, et al. [131]
miR-381-3p	Exogenous	Electroporation	Adipose-derived mesenchymal stem cells	Breast	MDA-MB-231 breast carcinoma cells	Inhibited proliferation, migration, and invasion capacity of cancer cells, as well as significant downregulation of genes and proteins related to the epithelial to mesenchymal transition that gives cancer cells increased motility	N/A	N/A	Shojaei, Samaneh, et al. [132]
miR-449a	Endogenous	Recombinant Plasmid Transfection	A549 human non-small cell lung cancer (NSCLC) cells	Lung	A549 human NSCLC cells	Inhibition of cell proliferation and promotion of apoptosis	Murine A549 xenograft model	Significantly decreased tumor volume and increased survival time of the treated mice	Zhou, Wen, et al. [120]
miR-126	Exogenous	ExoFectin®	MDA-MB-231 breast carcinoma cells	Lung	A549 human non-small cell lung cancer (NSCLC) cells	Higher internalization by A549 cells compared to human embryonic lung fibroblast cells and strongly suppressed A549 cell proliferation and migration	Murine A549 xenograft model	Successful lung homing after intravenous injection followed by inhibition of lung metastasis formation	Nie, Huifang, et al. [133]
miR-497	Exogenous	Transfection	HEK293T cells	Lung	A549 human NSCLC cells and human umbilical vein endothelial cells (HUVECs)	Suppression of tumor growth and downregulation of related genes. Additionally, in the 3D microfluidic system, a decrease of both endothelial cell tube formation and migration of tumor cells	N/A	N/A	Jeong, Kyeongsoo, et al. [134]
miR-375	Endogenous	Lipofectamine™ Transfection	Bone marrow-derived MSCs (BM-MSCs)	Liver	SNU-449 and Huh7 human HCC cell lines	Reduction of proliferation rate, elevated levels of apoptosis, and decrease in the number of migratory and invasive HCC cells	Murine Huh-7 xenograft model	Significant inhibition of Huh-7 tumor growth and reduction in the number of KI67-positive cells	Yu, Zhaoxia, et al. [135]
miR-125b	Both	Transfection	Huh7 human hepatocellular carcinoma (HCC) cells	Liver	SK-HEP1 adenocarcinoma and SNU-449 hepatocellular carcinoma cells	Inhibition of migration and invasion, downregulation of mRNA expression of MMPs -2, 9 and 14, disruption of TGF-ꞵ1-induced epithelial to mesenchymal transition, and SMAD2 protein expression	N/A	N/A	Kim, Hye Seon, et al. [136]
miR-451a	Endogenous	Lipofectamine™ Transfection	HUCMSCs	Liver	Hep3B and SMMC-7721 HCC cells	Reduction in cell survival rate and viability and promotion of apoptosis by downregulating ADAM10 gene expression	N/A	N/A	Xu, Yunxiuxiu, et al. [137]
miR-30a-3p	Exogenous	Lipofectamine™ Transfection	Human hepatic stellate cells LX-2	Liver	SK-Hep-1 and SMMC-7721 HCC cell lines	Inhibition of HCC cell migration, invasion and metastasis by targeting SNAP23	Murine SK-Hep-1 xenograft model	Fewer metastatic colonies	Liu, Chengdong, et al. [138]
miR-199a	Endogenous	Lentivirus Infection	Adipose tissue-derived MSCs	Liver	PLC/PRF/5 cells	Increased cancer cell sensitivity to doxorubicin and reduction in cell proliferation and viability	Murine PLC/PRF/5 xenograft model	Inhibition of tumor growth and increased effectiveness of doxorubicin	Lou, Guohua, et al. [139]
miR-34a	Exogenous	CaCl2 Transfection	CT-26 murine colon cancer cell line	Colorectal	CT-26 murine colon cancer cell line	Efficient delivery of functional miR-34a mimic	Murine CT-26 xenograft model	Significantly reduced tumor size and prolonged survival time. Induction of T cell polarization towards CD8 + T subtypes	Hosseini, Maryam, et al. [140]
miR-375-3p	Exogenous	CaCl2 Transfection	HT-29 and SW480 human colon cancer cell lines	Colorectal	T-29 and SW480 EMT-induced cells	Inhibition of cancer cell migration and invasion as well as reversed epithelial to mesenchymal transition via the downregulation of mesenchymal markers and upregulation of epithelial markers	N/A	N/A	Rezaei, Ramazan, et al. [141]
miR-124-3p	Exogenous	CaCl2 Transfection	CT-26 murine colon cancer cell line	Colorectal	CT-26 murine colon cancer cell line	Efficient delivery of functional miR-124-3p mimic	Murine CT-26 xenograft model	Significant tumor growth inhibition and increased median survival time	Rezaei, Ramazan, et al. [142]
miR-181c	Endogenous	Lentiviral Transduction	Bone marrow stromal cells	Ovarian	A2780 human ovarian cancer cell line and A2780/DDP, a DDP-resistant cell line	Suppression of cisplatin resistance in OC cells via downregulation of mesoderm-specific transcript and subsequent inactivation of the Wnt/ꞵ-catenin signaling pathway	Murine A2780/DDP xenograft model	Significant reduction in tumor growth, mass and volume	Ruan, Zhengyi, et al. [143]
miR-484	Exogenous	Electroporation	HEK293T cells	Ovarian	HUVEC cells	Inhibition of angiogenesis	Murine OVCAR-3-Luc xenograft model	Improved vascular normalization and sensitization of ovarian tumors to chemotherapeutics	Zhao, Zongxia, et al. [123]
miR-424	Endogenous	Lipofectamine™ Transfection	Bone marrow-derived mesenchymal stem cells from tibias and femurs of C57BL/6 mice	Ovarian	Normal human ovarian epithelial cell line (HOSEpiC), human ovarian cancer cell lines (SKOV-3, HO8910, A2780), and human umbilical vein endothelial cells (HUVECs)	Repress proliferation, migration, and invasion of ovarian cancer cells, reducing the expression of VEGF and VEGFR. Furthermore, inhibit the proliferation, migration, and tube formation of human umbilical vein endothelial cells	Murine SKOV-3 xenograft model	Suppressed tumorigenesis and angiogenesis	Li, Ping, et al. [144]
miR-494-3p	Exogenous	Electroporation	SKOV3 ovarian cancer cells	Ovarian	OVCAR3 and SKOV3 ovarian cancer cell lines	Reduction of OC cell growth and promotion of apoptosis	N/A	N/A	Wang, Suli, et al. [145]
miR-199a-3p	Exogenous	Electroporation	Fibroblasts derived from the normal omentum of patients	Ovarian	CaOV3, OVCAR3 and SKOV3 ovarian cancer cell lines	Inhibiton of cell proliferation and invasion	Murine SKOV3-13 xenograft model	Drastically inhibited peritoneal dissemination and reduced expression of c-Met expression, ERK phosphorylation, and MMP-2	Kobayashi, Masaki, et al. [146]
miR-124a	Endogenous	Lentiviral Transduction	BM-MSCs	Brain	Five Glioma stem cell lines (GSC267, GSC20, GSC6-27, GSC8-11, and GSC2-14)	Significant reduction in viability and clonogenicity of glioma stem cells	Mnurine intracranial GSC267 xenograft model	50% survival rate over a 120 day period, whereas both the PBS and unloaded-exosome treatments had a 0% survival rate by day 70	Lang, Frederick M., et al. [124]
miR-124	Exogenous	Lipofectamine™ Transfection	HEK293T cells	Brain	Human glioblastoma cell lines U373MG and U87MG and immortalized SV40 microglial cell line	Anti-tumor effects via the decreased expression levels of genes associated with tumor progression, as well as decreased M2 microglial polarization markers. Additionally, the use of the 3D microfluidics experiments showed decreased migration of both cell types, as well as increased tumor suppression via altered cytokine levels	N/A	N/A	Hong, Soohyun, et al. [147]
miR-100, anti-miR-21	Endogenous	Lipofectamine™ Transfection	Control neural stem cells (NSCs) and CXCR4- engineered NSCs	Brain	U87 glioma cells	Increased chemosensitization to temozolomide	Murine miRNA-loaded mpEVs intranasal delivery model	Increased glioblastoma cell sensitivity to the chemotherapeutic temozolomide, resulting in significant tumor regression and improved survival of the mice	Wang, Kai, et al. [148]
miR-744-5p	Endogenous	Lipofectamine™ Transfection	Human MSCs	Brain	U87 and U251 Human glioma cell lines	Delayed cell proliferative, invasive, and migration capabilities	Murine U87/THP-1 + PMA xenograft model	Reduced rate of cancer cell growth and reduction of tumor nodules in lung tissue	Liu, Ling, et al. [149]
miR-1252-5p	Exogenous	Electroporation	HEK293T cells	Multiple myeloma	RPMI-8226 B Lymphocyte cancer cells	Increased sensitization of RPMI-8226 cells to Bortezomib, leading to decreased cell viability	N/A	N/A	Rodrigues-Junior, Dorival Mendes, et al. [150]
miR-128-3p	Endogenous	Lipofectamine™ Transfection	HUCMSCs	Pancreatic	PANC-1 pancreatic carcinoma cells	Suppression of proliferation, invasion, and migration via targeting Galectin 3	N/A	N/A	Xie, X., et al. [151]
miR-142-5p	Endogenous	Lentiviral Infection	5-8F and CNE-3 nasopharyngeal carcinoma cells	Nasopharyngeal carcinoma	Radiotherapy-resistant 5-8F and CNE-3 nasopharyngeal carcinoma cells	Inhibition of cell proliferation and radioresistance	Murine 5-8FR xenograft model	Inhibition of tumor growth and radioresistance and acceleration of apoptosis in radiotherapy-resistant cells via inhibition of HGF/c-Met and EGF/EGFR pathways	Zhu, Changyu, et al. [152]
miR-144-3p	Endogenous	Plasmid Transfection/ Lentiviral Transduction	BM-MSCs	Cervical	SiHA squamous carcinoma cells	Suppression of cancer cell migration, invasion, proliferation, and colony formation	Murine SiHA xenograft model	Increased tumor cell apoptosis and significant decrease in tumor growth rate and size via downregulation of CEP55	Meng, Qin, et al. [153]
miR-371b-5p	Exogenous	Electroporation	HEK293T cells	Osteosarcoma	143B osteosarcoma, A549 lung carcinoma, and HeLa cervical cancer cells	Osteosarcoma cells took up loaded-EVs at a much higher rate as compared to other cancer cell types and showed significantly decreased cell viability while HeLA and A549 cell viability did not significantly change	Murine 143B xenograft model	Greatly decreased tumor volume as compared to unloaded exosomes and controls as well as significantly increased survival time of mice	Xue, Qiang, et al. [154]
miR-7-5p	Endogenous	Lipofectamine™ Transfection	BM-MSCs	Acute myeloid leukemia	HL60 and MOLM13 Myeloid Leukemia cells	Significantly decreased proliferation rate and increased apoptosis rate via downregulation of OSBPL11 gene	Murine MOLM13 xenograft model	Significantly decreased cancer load of splenic leukemia, increase in cancer cell apoptosis and decreased cancer cell proliferation	Jiang, Duanfeng, et al. [155]
miR-21	Exogenous	Electroporation	Blood plasma from mice	Heart	N/A	N/A	Murine Chemotherapy-related cardiotoxicity model	Reduction in cardiotoxicity after doxorubicin administration and restored cardiac function via increase in left ventricular ejection fraction and increased E wave/A wave ratio	Sun, Wenqi, et al. [156]

### Musculoskeletal disorders

Musculoskeletal disorders affect the motor organs, muscles, tendons, bones, cartilage, ligaments, and nerves, which are characterized by varying forms of discomfort to irreversible and disabling injury [157]. Moreover, the prevention and treatment of musculoskeletal disorders is a global concern, fueled primarily through government and private company efforts. More recently, in several classified musculoskeletal disorders, adipose-derived mesenchymal stromal cell EVs have been shown to improve tissue healing as a primary paracrine effector, providing yet another potential alternative therapeutic solution [158–162]. While the underlying mechanisms of EV-mediated repair are not explicitly and/or fully understood, studies entailing aspects of bone, intervertebral disc degeneration (IVDD), and cartilage therapies have begun to focus on aspects of deciphering EV-mediated cargo and subsequent RNA manipulation for the generation of miRNA-engineered EVs for downstream therapeutic applications.

Therapies involving miRNA-engineered EVs in bone tissue profile two main categories; (1) the treatment of osteoporosis and (2) attempts to increase osteogenesis for larger defects. In this respect, miRNA-loaded EV approaches using miR-19b-3p applied to osteoporotic BMMSCs resulted in the promotion of osteogenic differentiation and potential, evidenced by the significant upregulation of *ALP*, *collagen type 1*, and *RUNX2* expression, compared to the unloaded control exosome treatment [163]. Subsequent investigations of miRNA-loaded EV therapies in the intervertebral disc have followed a similar pattern in accordance to bone regenerative strategies, primarily using miRNA sequencing analysis techniques, as a means to identify potentially therapeutic miRNAs. Indeed, Zhang et al. identified a potential candidate miRNA by first recognizing six major proteinases that drive IVDD. Through functional use of IL-1β as a means to mimic degenerative conditions, miR-27a-loaded EVs were applied to degenerated rat nucleus pulposus cells and showed significant protein alterations, such as upregulated collagen type II and aggrecan, and conversely correlated decreases in their potent degradative molecule, MMP-13 [164]. Apart from IVDD, investigations involving cartilage tissue have almost exclusively honed in on the alleviation of osteoarthritis (OA), a habitual disease affecting the joints. Using similar approaches to those aforementioned, multiple groups have focused on miRNA-loaded EV strategies in orthopaedic tissue through the comparison of miRNA expression profiles of normal and OA-afflicted tissues. In vitro, exosomes from synovial fibroblasts isolated from knee joints of Sprague–Dawley rats loaded with miR-126-3p, have been shown to decrease chondrocyte apoptosis and suppress inflammation through decreased levels of IL-1β, IL-6, and TNF-α. Furthermore, using an OA rat model in vivo, miR-126-3p-loaded exosomes were also shown to decrease the occurrence of abnormal lesion- or edema-like inflammation, restore bone volume fraction levels, and to increase the articular cartilage thickness and surface regularity [165]. Nevertheless, the breadth of studies in this regard (Table 3) contain a wide-array of interfacial engineering strategies that require extensive investigation in further developing this technology, as both a promising diagnostic and an effective therapeutic system.

**Table 3:** miRNA-engineerd EVs as an advanced drug delivery system in *Musculoskeletal Disorders*

Therapeutic miRNA	miRNA Enrichment	Incorporation method	Source of EVs	Target	in vitro Cell Lines Utilized	in vitro Findings	in vivo Model	in vivo Findings	Source
miR-19b-3p	Exogenous	HiPerfect® Transfection	Serum from 1-month-old rats	Osteoporosis	Osteoporotic BM-MSCs	Higher osteogenic potential through increased expression of osteogenic genes (ALP, collagen type I, and RUNX2) and greatly increased collagen type 1 expression	N/A	N/A	Xun, Jingqiong, et al. [166]
miR-101	Endogenous	Lentiviral Transduction	Human BMSCs (hBMSCs)	Osteoporosis	hBMSCs	Increase in both osteogenic activity and osteogenic differentiation of MSCs as demonstrated by increased alkaline phosphatase activity and expression of osteogenic markers RUNX2, Osterix, OCN, and osteopontin	N/A	N/A	Li, Yanhong, et al. [167]
miR-935	Endogenous	Lipofectamine™ Transfection	hBMSCs	Osteoporosis	hFOB1.19 human osteoblast cells	Improved osteoblast cell proliferation and differentiation as seen through increased RUNX2 and activating transcription factor 4 protein levels	N/A	N/A	Zhang, Ying, et al. [168]
miR-20a	Endogenous	Lipofectamine™ Transfection	hBMSCs	Osteoporosis	hBMSCs	Successful promotion of in vitro migration and osteogenesis of BM-MSCs as seen through upregulation of ALP, RUNX2, and OCN genes	Rat osteoporosis model	Significantly increased bone volume fraction, total bone volume, and bone mineral density	Liu, Wei, et al. [169]
miR-27a	Endogenous	riboFECT Transfection	Autophagy Activated Nucleus pulposus cells (NPCs)	Intervertebral disc degeneration (IVDD)	NPCs	Protein analysis indicated significantly upregulated collagen type II and aggrecan levels, coinciding with a significant decrease in MMP-13 levels	N/A	N/A	Zhang, Qi‐Chen, et al. [164]
miR-129-5p	Endogenous	Lipofectamine™ Transfection	hBMSCs	IVDD	Human NPCs	Increased cell viability combined with increased collagen type II and aggrecan production	Rat IVDD model	Successfully inhibited M1 polarization, reversed cellular apoptosis and extracellular matrix degradation	Cui, Shaoqian, and Lei Zhang. [170]
miR-15a	Endogenous	Lipofectamine™ Transfection	Human NP-MSCs	IVDD	Dedifferentiated MSCs from human NP tissue	Significantly increased MSC proliferation and colony formation as well as increased MSC chondrogenic differentiation shown through upregulation of aggrecan and collagen type II mRNA levels coupled with the downregulation of multiple proteinase mRNAs	N/A	N/A	Zhang, Qiang, et al. [171]
miR-17-5p	Endogenous	Lipofectamine™ Transfection	BMSC-bm	IVDD	Human NP cells	Increase in human NP cell proliferation, decrease in apoptosis rate, and significant increase in collagen type II and aggrecan protein levels coupled with significantly decreased MMP-13 and ADAMTS5 levels	Rat IVDD model	Similar changes in protein levels to in vitro studies, coupled with lower histological grading score of IVDD	Zhou, Zhi-Min, et al. [172]
miR-125b-5p	Endogenous	Lipofectamine™ Transfection	Cartilage endplate stem cells	IVDD	Tert-buty l hydroperoxide (TBHP) induced NPCs	Inhibited the apoptosis of TBHP-NPCs as well as the expression of bax, MMP13, and p62 while increased the expression of bcl2, ACAN, and LC3-II/I	Rat IVDD model	Upregulation of BCL2 apoptosis regulator, ACAN, and LC3-II/I coupled with the downregulation of SUV39H1 histone lysine methyltransferase, bax, MMP-13, and ubitquitin-binding protein p62. Histological analysis revealed decreased NP cell apoptosis and reduced fibrotic remodeling of the NP	Chen, Dong, and Xin Jiang. [173]
miR-126-3p	Endogenous	Transfection	Synovial fibroblasts	Osteoarthritis (OA)	Rat chondrocytes	Decrease in apoptosis in chondrocytes and suppression of their inflammation as seen through decreased IL-1b, IL-6, and TNF-a	Rat OA model	Anti-apoptotic and anti-inflammatory effects on cartilage, suppression of osteophyte formation, decreased occurrence of abnormal lesion- or edema-like inflammation, restoration of bone volume fraction levels, and increased articular cartilage thickness and surface regularity as seen through histological analysis	Zhou, Yan, et al. [174]
miR-361-5p	Exogenous	Electroporation	hBMSCs	OA	IL-1β-treated chondrocytes	Significantly increased cell viability and downregulation of proteinases involved in OA progression	Rat OA model	Downregulation of proteinases involved in OA as well as increased synovial tissue hyperplasia and decreased OA pathological scoring	Tao, Yunxia, et al. [175]
miR-140	Exogenous	Electroporation	Human Chondrocytes	OA	IL-1β-treated chondrocytes	Downregulated MMP-13 and ADAMTS-5 gene expression	Rat OA model	Downregulation of MMP-13 and ADAMTS-5, while cartilage tissue repair was nearly identical to the sham surgical group as evidenced by histological scoring	Liang, Yujie, et al. [176]
miR-140	Exogenous	Freeze and Thaw Method	Rabbit serum	Cartilage regeneration	Rabbit BM-MSC and chondrocytes	Exhibited chondrogenic differentiation through changes in BM-MSC morphology, as well as the upregulation of collagen type II, Sox9, and ACAN genes in BM-MSCs	N/A	N/A	Won Lee, Gi, et al. [101]
miR-29a-3p	Endogenous	Agonist	HUCMSCs	Tendon regeneration	N/A	N/A	Rat achilles tendon defect model	Significantly increased expression of tendon markers TNMD and SCXA, elevated COL1A1 gene expression and higher average ultimate tensile strength, stiffness, and Young's modulus in treated tendons	Yao, Zhixiao, et al. [177]
miR-24	Endogenous	Lentiviral Transduction	HMSCs	Bone repair	HMSCs, macrophages	Enhanced osteoinductive function by activating SMAD1/5/8 phosphorylation and MSC differentiation	Rat calvarial bone defect model	Enhanced bone repair and anti-inflammatory activity	Huang CC, et al. [178]

### Cardiovascular disorders

Accounting for almost a third of all deaths, cardiovascular disease is the leading, predominant, and principal underlying cause of death worldwide [179]. Aside from established cardiac care and continued advancements in certain aspects of applied research, calls for novel and strategic alternatives, such as therapeutically-enriched, miRNA-engineered EVs, are imperative in pushing the threshold on the forefront of nanomedicine. Initially emerging as a promising diagnostic tool, the noninvasive nature of EV collection has propagated their exploitation for disease prevention, intervention, and palliation in cardiovascular disease and repair [180]. Repair strategies for the heart entailing miRNA-engineered EV therapies have largely focused on aspects of regeneration post-heart attack, either through the investigation of ischemia–reperfusion (I/R) injury or via myocardial infarction. As such, parameters for healing in these models are generally characterized by reduced inflammation and rates of apoptosis (Table 4). Hence, Chen et al. showed that in vitro, miR-125b loaded exosomes applied to rat myocardium cells derived from an I/R injury model led to the restoration of cell viability (similar values to the sham rat myocardium cells), as well as decreased apoptosis and levels of inflammatory proteins IL-1β, IL-6 and TNF-α. Moreover, the same group showed that in vivo, the cardiac function of I/R rats was restored following the administration of miR-125b loaded exosomes through lowered infarct size, reduced ratios of inflammatory cells, and restored left ventricular function [181]. Collectively, this suggests in part, the role of miR-125b in contributing to the remission of I/R in myocardium and its functional applicability to serve as a potential therapeutic agent for myocardial I/R. MicroRNA-engineered EV therapies for cardiovascular repair have thus far shown in vivo promise for the treatment of several prominent and deadly cardiac pathologies, however, additional work is needed to exclusively translate these therapeutic applications bedside for the implementation in human patients.

**Table 4:** miRNA-engineerd EVs as an advanced drug delivery system in *Cardiovascular Disorders*

Therapeutic miRNA	miRNA Enrichment	Incorporation method	Source of EVs	Target	in vitro Cell Lines Utilized	in vitro Findings	in vivo Model	in vivo Findings	Source
miR-125b	Endogenous	Lipofectamine™ Transfection	Rat BM-MSCs	Ischemia reperfusion (I/R) injury model	Rat I/R myocardium cells	Restoration of cell viability coupled with a decease in the apoptotic ratio. Additionally, the levels of IL-1b, IL-6 and TNF-a proteins were significantly reduced, indicating reduced cellular inflammation	Rat Ischemia reperfusion (I/R) model	Restored cardiac function as seen through significantly lower infarct size, reduced ratio of inflammatory cells, and restored left ventricular function	Chen, Qi, et al. [181]
miR-494-3p	Endogenous	RiboFECTTM Transfection	Murine bone marrow-derived dendritic cells	Murine myocardial infarction	Cardiac microvascular endothelial cells	Enhanced tube formation	Murine myocardial infarction model	Increased VEGF expression and a significant increase in the mean number of vessels in the myocardium	Liu, Haibo, et al. [182]
miR-210	Endogenous	Transfection	Human Adipose Derived Stem Cells	Ischemia heart disease	Hypoxic endothelial cells and H9C2 cardiomyocyte cells	Inhibition of EFNA3 in endothelial cells and a significant increase in VEGF expression combined with an increase in viability for cardiomyocyte cells	Rat I/R injury model	Significant increase in cardiac function and no significant changes in fibrotic area ratio	Song, Byeong-Wook, et al. [183]
miR-144-3p	Exogenous	Exo-Fect™ Transfection	HEK293 cells	Murine myocardial infarction	H9C2 rat myoblast cells	Increased cell viability, decreased lactate dehydrogenase release, and decreased cellular apoptosis after H2O2-induced cellular injury	Murine myocardial infarction model	Reduction of infarct size, significantly reduced cell death, and no systemic toxicity	Kang, Ji-Young, et al. [184]
miR-126	Endogenous	Lipofectamine™ Transfection	Human BM-MSCs	Nonspecific angiogenesis	HUVECs	Enhanced proliferation, migration, and tube formation	Murine full-thickness cutaneous wound model	Significantly increased wound closure rate, reduction of scar formation, and enhancement of angiogenesis	Zhang, Lei, et al. [185]
miR-186	Endogenous	Lipofectamine™ Transfection	Human BM-MSCs	Idiopathic pulmonary fibrosis	LL29 human lung fibroblasts	Suppression of lung fibroblast activation and reduced fibroblast viability and invasion	Murine pulmonary fibrosis model	Reduced expression of myofibroblastic markers, reduction in collagen fibers, impaired fibroblast activation, and suppression of idiopathic pulmonary fibrosis	Zhou, Jing, et al. [186]
miR-146a, miR-370, miR-126a	Endogenous	Trans-IT TKO (Mirus)	NHDF-EVs	Ischemic heart disease	C57BL/6 mouse primary neonatal cardiomyocytes	Therapeutic miRNA cluster acts synergistically to reduce cardiomyocyte apoptosis	N/A	N/A	Euscher LM, et al. [187]

### Neurological disorders

The brain is an infinitely complex, temporally, and spatially multiscale structure that is far from well understood. The development of new treatments and therapies for brain-related diseases are furthermore hindered due to epithelial-like tight junctions within the brain capillary endothelium that compose the blood–brain barrier (BBB), preventing the uptake of most pharmaceutical compounds [188]. Fortunately, EVs effectively traverse this restriction via the bidirectional BBB transendothelial transport, both from the blood into the brain and from the brain into the blood [189]. Hence, treatments for neurodegenerative afflictions using miRNA-engineered EVs compose a strategic and novel system to combat ailments like subarachnoid hemorrhage, alzheimer’s disease, and depression (Table 5).

**Table 5:** miRNA-engineerd EVs as an advanced drug delivery system in *Neurological Disorders*

Therapeutic miRNA	miRNA Enrichment	Incorporation method	Source of EVs	Target	in vitro Cell Lines Utilized	in vitro Findings	in vivo Model	in vivo Findings	Source
miR-194	Endogenous	Lentiviral Infection	Murine BM-MSCs	Neurovascular endothelial cell injury	Human brain microvascular endothelial cells	Restoration of cell viability and migratory ability after oxygen–glucose deprivation/reoxygenation treatment and inhibition of ferroptosis	N/A	N/A	Li, Xu, et al. [190]
miR-223-3p	Endogenous	Lentiviral Infection	Murine BM-MSCs	Cerebral ischemic injury	Murine BV-2 microglia cells	Promotion of M1 to M2 phenotypic conversion of microglia cells as well as modulation of inflammatory cytokine expression, indicating a shift towards an anti-inflammatory remodeling phenotype	Rat middle cerebral artery occlusion and reperfusion model	Improved neurological dysfunction and decreased infarct volume over a 28-day period	Zhao, Yangmin, et al. [191]
miR-17-5p	Endogenous	Lipofectamine™ Transfection	Rat primary astrocytes	Hypoxic-ischemic brain damage	Immortalized H19-7 microglial cell line	Promotion of cell viability and reduced inflammation after OGD-induced damage	Neonatal rat model of hypoxic-ischemic brain damage	Treatment alleviated brain damage and coincided with high expression of miR-17-5p. Additionally increased the healing by reducing neuronal apoptosis and inflammation	Du, Lin, et al. [192]
miR-210	Endogenous	DharmaFECT Transfection	Human endothelial progenitor cells	Ischemia-induced neuronal damage	SH-SY5Y human neuroblastoma cells	Reduced hypoxia and reoxygenation (H/R) induced apoptosis, attenuation of intracellular reactive oxygen species levels, and increased neuroblastoma proliferation	N/A	N/A	Yerrapragada, Sri Meghana, et al. [193]
miR-193-3b	Exogenous	Electroporation	Murine BM-MSCs	Neuroinflammation following subarachnoid hemorrhage	N/A	N/A	Murine subarachnoid hemorrhage (SAH) model	Reduced inflammatory cytokine levels, improved neurological scoring, and decreased brain edema after SAH treatment, which indicates alleviation of neurobehavioral impairments	Lai, Niansheng, et al. [194]
miR-22	Endogenous	HiPerfect® Transfection	Mouse Adipose-derived MSCs	Alzheimer’s disease	PC12 rat adrenal medulla cells	Inhibition of apoptosis and significantly decreased release rate of inflammatory factors	Murine APP/PS1 model	Increased nerve function and motor ability, improved survival of nerve cells, and decreased expression of inflammatory factors	Zhai, Liping, et al. [195]
miR-26a	Endogenous	Lipofectamine™ Transfection	Human BM-MSCs	Depression	Neonatal rat hippocampal neurons	Elevated superoxide dismutase levels and inhibition of inflammatory factors in hippocampal neurons as well as increased hippocampal neuron proliferation and restriction of apoptosis	N/A	N/A	Guo, Huirong, et al. [196]
miR-146a	Endogenous	Lipofectamine™ Transfection	Murine BM-MSCs	Diabetic peripheral neuropathy	Human dermal microvascular endothelial cells	Inhibition of inflammatory activation	Murine diabetic peripheral neuropathy model	Significantly increased intraepidermal nerve fiber density, restoration of nerve fiber diameter, increased axonal remyelination, and inhibition of inflammatory effects	Fan, Baoyan, et al. [197]
miR-494	Exogenous	Exo-Fect™ Transfection	Rat BM-MSCs	Spinal cord injury	Rat dorsal root ganglion cells and alveolar macrophages	Significantly increased ganglion cell viability and promotion of M2 macrophage polarization	Rat spinal cord injury model	Homing to spinal cord lesion, reduction of lesion volume, regeneration of neurofilaments, and recovery of rat behavioral function	Huang, Wei, et al. [198]

Implementation of miRNA-engineered EVs to treat brain illnesses inherently provoke query on their capacity to reach targets in effective concentrations after systemic/intranasal administration, or targeted deposition. Comparing the global miRNA profiles from plasma exosomes of subarachnoid hemorrhage (SAH) and healthy patients, followed by extrapolation to a murine model of SAH, Lai et al. functionally revealed the role of miR-193-3b-loaded BM-MSC-derived exosomes in reducing inflammatory cytokine levels, improving neurological scoring, and decreasing brain edema post-SAH treatment, all of which indicate neurobehavioral impairment alleviation [199]. This study, along with others denoted previously, precisely specify the value of using multi-omics analysis to accurately identify differentially regulated miRNAs that govern health and disease. Such studies unveiling the functional applicability of miRNA-engineered EVs using small animal models suggest their preeminent role as future clinical therapeutic treatments. Additionally, at minimum, rigorous and in-depth studies are still needed to optimize various aspects of EV production, loading, and administration, prior to potential clinical EV-based therapy commercialization.

## Therapeutic use of miRNA-loaded EVs under clinical trial

EV therapies have been investigated amid multiple clinical trials, demonstrating acceptable safety profiles and therapeutic proof of efficacy in humans [200–203]. While promising, at least 1 miRNA-engineered EV therapy exists under clinical trial, although its results have yet to be substantiated and published. Additionally, phase I/II testing of allogenic MSC-derived exosomes loaded with miR-124 for the treatment of acute ischemic stroke is also currently in the recruiting phase, with the trial expected to consist of 5 patients (NCT03384433). Aside from EVs, several miRNA-based therapies have already undergone and completed clinical trials. In the past several years, in total, at least 4 miRNAs (miR-16, miR-29, miR-34a, and miR-124) have shown clinical relevance for subsequent translation to EV-based therapeutics.

A multicenter phase 1 clinical trial completed several years back used miR-16 to treat mesothelioma and non-small cell lung cancer (NCT02369198). Twenty-six pleural mesothelioma patients received weekly doses of miR-16 loaded into non-living bacterial minicells (TargomiRs), which revealed an acceptable safety profile, producing an objective response in 1 of 22 patients. The foremost side-effect observed was a heightened inflammatory response, although unable to pinpoint and decipher its origins as the delivery of miR-16, the bacterial origin of the TargomiRs, or an antitumor effect [204, 205]. Aside, the confirmation of an acceptable safety profile is conclusively promising for miR-16’s clinical use, which has also shown effects in the attenuation of lung inflammation and the reduction of lung injury in mice, when targetly delivered via ADSC-derived exosomes [206]. Moreover, a phase 1 clinical trial was also conducted on MRG-201, a synthetic drug designed to mimic the bioactivity of miR-29 (NCT02603224). In total 54 healthy volunteers were enrolled to the study and assigned to either intact or incised skin groups. Volunteers received intradermal injections of either a single or multiple doses of MRG-201, considered safe and well-tolerated at all levels, with a total of 139 doses given to 47 subjects. Collectively, it was shown that MRG-201 treatment decreased wound fibroplasia with no evidence of wound dehiscence [207]. Efficacy and tolerance of MRG-201 permits significant credibility to the clinical potential of miR-29-engineered-EVs, which have recently shown promising results as a strategic effort in tendon regeneration [177].

To date, miR-34a delivered intravenously to patients with solid tumors refractory to standard treatment has been investigated within two phase 1 clinical trials (NCT01829971 and NCT02862145). The first trial aimed to establish and optimize miR-34a dosing associated with its acceptable safety. One patient with HCC evidenced the confirmation of a prolonged response (at minimum a 30% decrease in the longest diameter sum of the target lesions), while four others experienced the persistence of stable disease (neither sufficient shrinkage, nor sufficient increase in the longest diameter sum) [208]. The second trial was abruptly closed early due to serious immune-mediated adverse events within four patients, yet demonstrated a manageable toxicity profile in the majority [209]. These studies corroborate the proof-of-concept required in establishing miR-34a therapeutics, while engineered-EV treatments have been recently studied in light of breast [119], and colorectal [122] cancer suppression. Alas, a phase IIa clinical trial has also been conducted on ABX464, an orally administered small molecule that induces the splicing of miR-124 (NCT03093259). A total of 32 participants with moderate to severe ulcerative colitis were recruited to the study and enrolled in an 8-week induction phase, followed by an optional long-term extension phase. ABX464 was shown to be safe and well-tolerated, while also greatly increasing both the clinical remission and response amid the treatment group over the initial 8-week period. During the extension phase, a high maintenance of remission rates persisted, with the majority of patients remaining in clinical remission to the 12 and 24-month time points. Currently, the safety and efficacy of ABX464 is ongoing and being further investigated within a phase IIb clinical trial, with 254 recruited participants (NCT03760003). Preliminary results aid in the promising perspective for miR-124-engineered-EV therapeutics, which have duly recently been investigated in glioblastomas [124, 147], colorectal cancer [210], and spinal cord ischemia–reperfusion injury [211] treatment. Taken together, the clinical evaluation of miRNA-engineered EV therapies is still in its relative infancy. However, with one therapy under clinical trial thus far, and multiple successful proposed examples of miRNA-loaded EV strategies published, it is highly likely that the number of proposed miRNA-engineered EVs for therapeutic use will steadily rise within the next decade.

## miRNA loaded EVs: limitations hindering clinical progression in nanomedicine

**Fig. 4:**
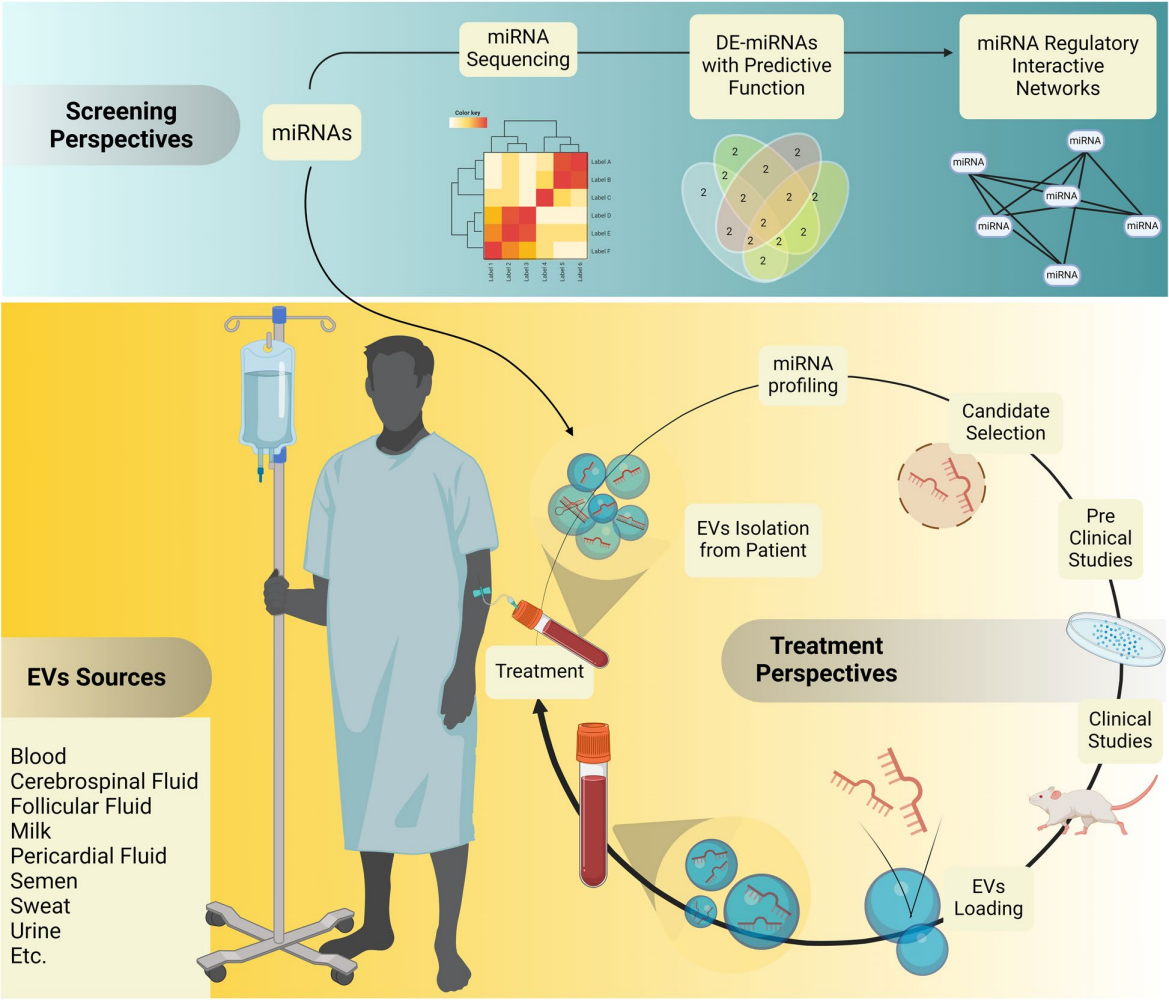
Treatment Perspective of EV-miRNAs for Clinical Use as Therapeutics. EVs carrying host molecules, readily isolated from systemic fluids, propagate EVs as an effective screening perspective or diagnostic tool in the identification of biomarkers for many diseases and their potential application as an effective therapeutic treatment

Outlined in Fig. 4, the premise to move miRNA-engineered EV therapeutics from benchtop and small animal models, to clinical trials is a time and resource-intensive process. In order to produce a potential therapeutic treatment for clinical testing, standards in EV purity must be met. In addition, such therapeutics require the isolation of a large number of EVs, underscoring the need for reproducible and scalable methods. To meet purity standards set forth for EV-based treatments, several methodologies have been developed to meet Good Manufacturing Practices (GMP), a system of processing, standardized procedures, and documentation that establishes quality standards. This includes GMP strategies for the production and isolation of EVs from MSCs [212–214], HEK293 human embryonic kidney cells [215], and cardiac progenitor cells [216]. Established methodologies have helped to lay the groundwork for EV-based therapeutics in achieving GMP standards, not only in terms of isolation and purification, but duly in their scale-up production. To further scale-up the production of EVs in a controlled manner, a number of bioreactor systems have been developed [217–219], which have resulted in high-yields of EVs, while also serving as a platform to mechanically or chemically stimulate cells, as a means to augment the cargo of EVs. A full review was recently published focusing on large-scale cell culture platforms to increase EV yields, which further details the use of scale-up strategies currently being implemented in clinical trials [220].

Several groups have also attempted to generate clinically relevant numbers of EVs through the isolation of common biologics such as blood and milk. These methods drastically reduce the amount of time needed to obtain large amounts of EVs, removing the production time necessary to expand large quantities of cells in vitro. In addition, this method also avoids the need in standardizing cell culture strategies, by instead utilizing pre-existing GMP protocols for the standardized collection of biologics. Large-scale EV isolation from blood has been developed by several groups, either through the isolation of large quantities of cells to rapidly farm EVs or through direct isolation of the EVs themselves. A novel method developed to induce rapid EV production over a 48-h window has duly been established via isolating natural killer cells from 30-50 mL of donor blood [221]. Alternatively, direct isolation of EVs from blood plasma has equitably demonstrated high EV yields and purities [222]. Moreover, timely isolation methods also exist that were originally designed for the rapid and efficient isolation of EVs for proteomic analysis, which yield ~ 1*10^11^ EVs per milliliter of serum in 15 min, a promising method to generate clinically relevant numbers of EVs to be successfully scaled-up or scaled-out [223]. Combining established large-scale EV isolation methods from blood with the partnerships of blood banks and hospitals, further the applicability of such methods in collecting large quantities of GMP-grade EVs in the advancement of future clinical trials. On the other hand, milk-derived exosomes are even more readily available offering an alternative promising source of GMP-grade EVs for clinical trials. Marsh et al. developed protocols for the scalable production of EVs from bovine milk, creating ultra-dense isolates of sEVs that accounted for 10–15% of the total starting milk volume, resulting in incredibly high concentrations of EVs isolatable from an additional common biologic [224]. The use of milk-derived EVs for miRNA delivery have also recently been investigated, finding that hsa-miR148a-3p, can be successfully loaded into raw bovine milk derived-exosomes with confirmed uptake by hepatic and interstitial cell lines [225]. Although miRNA loading into milk-derived EVs has not yet been widely investigated, such studies effectively outline an additional, potentially promising, strategy to propagate miRNA-loaded EV therapies on a large scale that caters to satisfactory purities and reproducibility standards. Albeit therapeutically promising studies exist and in part, some of the prominent hurdles are currently being overcome, plausible concern still remains regarding the efficacy/dose optimization, biodistribution, and the target site bioavailability and engagement, which all require an increased and more developed understanding of EV biology prior to wide-scale clinical implementation.

## Concluding remarks and future perspectives

Over the past decade compounding knowledge of the structure of EVs, as well as their biogenesis and function, have catapulted advancements for their novel potential in pharmaceuticals as a next-generation drug delivery system. Functional studies denoting the roles of EV-coupled miRNAs continue to accumulate aiding in their incorporation in preclinical and clinical settings alike. Given the complex effects of miRNAs in post-transcriptional gene regulation, in combination with the additonal ambiguities that undermine their precise functions, a need to further elucidate such systems both in vitro and in vivo still persist. Moreover, predetermined mechanistic approaches to modify EVs for the incorporation of therapeutic miRNAs requires further technical optimization in managing dosage and other pharmacokinetic factors, prior to their large-scale distribution as a new frontier drug delivery platform. Further advancements in EV biology and the mechanistic approaches to effectively modify EVs with repeatability will favor the clinical translation of miRNA-engineered EVs for precise therapeutic application in treating a number of disease pathologies, continuing to push their implementation as novel payers on the forefront of nanomedicine.

## Data Availability

No datasets were generated or analysed during the current study.

## Abbreviations

EV: Extracellular vesicle
miRNA: MicroRNA
ISEV: International society for extracellular vesicles
MV: Microvesicle
ApoBD: Apoptotic body
MVB: Multivesicular body
ILV: Intraluminal vesicle
LAMP: Lysosome-associated membrane proteins
ESCRT: Endosomal sorting complex required for transport
MISEV: Minimal information for studies of extracellular vesicles
pri-miRNA: Primary miRNA
pre-miRNA: Precursor miRNA
siRNA: small interfering RNA
RBP: RNA-binding protein
HBS: HEPES-buffered saline
MSC: Mesenchymal stem cell
HCC: Hepatocellular carcinoma
CRC: Colorectal cancer
OC: Ovarian cancer
IVDD: Intervertebral disc degeneration
OA: Osteoarthritis
I/R: Ischemia-reperfusion
BBB: Blood brain barrier
SAH: Subarachnoid hemorrhage
GMP: Good manufacturing practices
